# The global governance of human cloning: the case of UNESCO

**DOI:** 10.1057/palcomms.2017.19

**Published:** 2017-03-21

**Authors:** Adèle Langlois

**Affiliations:** 1 University of Lincoln, Lincoln, Lincolnshire, UK

## Abstract

Since Dolly the Sheep was cloned in 1996, the question of whether human reproductive cloning should be banned or pursued has been the subject of international debate. Feelings run strong on both sides. In 2005, the United Nations adopted its *Declaration on Human Cloning* to try to deal with the issue. The declaration is ambiguously worded, prohibiting “all forms of human cloning inasmuch as they are incompatible with human dignity and the protection of human life”. It received only ambivalent support from UN member states. Given this unsatisfactory outcome, in 2008 UNESCO (the United Nations Educational, Scientific and Cultural Organization) set up a Working Group to investigate the possibility of a legally binding convention to ban human reproductive cloning. The Working Group was made up of members of the International Bioethics Committee, established in 1993 as part of UNESCO’s Bioethics Programme. It found that the lack of clarity in international law is unhelpful for those states yet to formulate national regulations or policies on human cloning. Despite this, member states of UNESCO resisted the idea of a convention for several years. This changed in 2015, but there has been no practical progress on the issue. Drawing on official records and first-hand observations at bioethics meetings, this article examines the human cloning debate at UNESCO from 2008 onwards, thus building on and advancing current scholarship by applying recent ideas on global governance to an empirical case. It concludes that, although human reproductive cloning is a challenging subject, establishing a robust global governance framework in this area may be possible via an alternative deliberative format, based on knowledge sharing and feasibility testing rather than the interest-based bargaining that is common to intergovernmental organizations and involving a wide range of stakeholders. This article is published as part of a collection on global governance.

## Introduction

UNESCO (the United Nations Educational, Scientific and Cultural Organization) was founded in 1945, aiming to “build peace in the minds of men” through education, science, culture and communication (UNESCO, 2007). Its Bioethics Programme began in 1993. The organization deems itself uniquely placed to lead the way in setting bioethical standards, as the only UN agency with a mandate for both the human and social sciences (UNESCO, 2016e). To this end, it has adopted three declarations on bioethics: the 1997 *Universal Declaration on the Human Genome and Human Rights* (UNESCO, 1997), the 2003 *International Declaration on Human Genetic Data* (UNESCO, 2003) and the 2005 *Universal Declaration on Bioethics and Human Rights* (UNESCO, 2005b). After drafting three declarations in the space of a decade, UNESCO decided to take a “normative pause” and instead focus on fostering take-up of the existing declarations regionally and nationally (UNESCO, 2005a). Before long, however, it started to consider a fourth bioethics instrument, an international convention on human cloning. From 2008 to 2011 it investigated whether an international convention to ban human reproductive cloning is warranted. The Working Group assigned to this question “flip-flopped” back and forth: in 2008 it recommended a convention, in 2009 it decided continued international dialogue would be sufficient and in 2010 it went back to a convention. As member states could not agree on a way forward, the issue was dropped in 2011 without a firm decision being made on the need or otherwise for a convention. This can be seen as a global governance failure. In 2014, the Bioethics Programme began to revisit the issue. This time there was greater consensus on the need for a ban on human reproductive cloning, but no practical progress has been made.

This article takes a traditional global governance scenario—a debate within a UN agency about whether to draft an international convention—and asks why the outcome was unsatisfactory. The analysis draws on first-hand observations of UNESCO’s publicly held bioethics meetings in 2010 and 2011, official UNESCO records of these and other meetings and UNESCO reports on human cloning. After a brief introduction to (a) developments in global governance and (b) the science and ethics of human cloning, the article charts the progress and ultimate collapse of the UNESCO cloning debate from 2008 to 2011 and developments from 2014 onwards. It concludes that, although human reproductive cloning is a challenging subject, establishing a global governance framework in this area may be possible via an alternative deliberative format.

## Global governance

Ruggie (2014: 5) defines governance as “systems of authoritative norms, rules, institutions, and practices by means of which any collectivity, from the local to the global, manages its common affairs”. At the global level these systems, particularly within formal intergovernmental settings such as UNESCO, are increasingly seen to be inadequate, with scholars variously describing them as “facing a deep crisis” (Pauwelyn *et al.*, 2014: 737), “suboptimal” (Ruggie, 2014: 15) and suffering the “pathologies” of gridlock, fragmentation, disconnect between related issue areas and conflicts of interest (Pegram and Acuto, 2015: 586). The old, hierarchical model of multilateral governance is considered too rigid (Pauwelyn *et al.*, 2014: 737) and to have “limited utility in dealing with many of today’s most significant global challenges” (Ruggie, 2014: 8). Traditional intergovernmental organizations have not adapted to the increasing complexity of society and the ensuing need for flexible regulatory mechanisms that can keep pace with scientific development (Pauwelyn *et al.*, 2014: 742–743).

These problems have led to changes and innovations in both the theory and practice of global governance (Ruggie, 2014; Weiss and Wilkinson, 2014; Pegram and Acuto, 2015: 588). As Pauwelyn *et al.* (2014: 734) note, “Formal international law is stagnating in terms of both quantity *and* quality. It is increasingly superseded by ‘informal international lawmaking’ involving new actors, new processes, and new outputs”. They refer to this stagnation as “treaty fatigue” (Pauwelyn *et al.*, 2014: 739). The international system is becoming more pluralist and less dominated by sovereign states pursuing narrow interests. There has been movement towards voluntary rather than binding regulation, as well as capacity building (Pauwelyn *et al.*, 2014: 736; Pegram and Acuto, 2015: 591). Particularly for emerging areas, such as the internet, regulation has been informal, with no discussion of a legally binding treaty (Pauwelyn *et al.*, 2014: 738). In turn, a “second generation” of global governance scholarship, which recognizes the complexity of global governance in a changed global context, is focusing less exclusively on intergovernmental politics. In the introduction to their special issue of *Millennium* on global governance’s “interregnum”, Pegram and Acuto (2015: 586 and 588) predict a “more innovative global governance research and practice-oriented agenda” and a transition to “a potentially more pluralist (and hopefully more democratic) intellectual and practical ecosystem, as well as to new structures of power”. This article applies some of these new practices and ideas to UNESCO’s human cloning debate, answering Pegram and Acuto’s call for “more empirical research” (Pegram and Acuto, 2015: 595).

## Human cloning and its current international regulation

Although the idea of human cloning excites strong views, there is much confusion about what it would actually entail. Cloning can take two forms: “reproductive” cloning and “therapeutic” or “research” cloning. These terms are not scientifically accurate, but are commonly used nevertheless. They stem from the process of somatic cell nuclear transfer, whereby an enucleated egg receives a nucleus from a somatic (body) cell. In reproductive cloning, the embryo is implanted into a female for gestation. Through this method, Dolly the Sheep became the first mammal to be cloned in July 1996. In therapeutic cloning, an embryo is harvested for stem cells rather than brought to term (Wilmut *et al.*, 1998: 21; Bowring, 2004: 402–403; Isasi *et al.*, 2004: 628; United Nations University Institute of Advanced Studies, 2007: 6). Although therapeutic cloning is held by many to have great potential medically, as a source of compatible tissue and organs for those who need transplants, it generates considerable controversy. For people who see human life as beginning at fertilization, therapeutic cloning is also reproductive (Isasi *et al.*, 2004: 628; Lo *et al.*, 2010: 17).

Since the cloning of Dolly the Sheep, ethicists, lawyers and scientists have argued vigorously both for and against developing this technology for use in humans. Those in favour draw on liberal values, citing reproductive freedom, or hope that cloning will provide a new means to tackle infertility. Those against fear for the psychological health of the clone, who would be unable to enjoy what they see as the inherently human quality of having a unique identity. Clones might be expected by their “parents” to conform to a particular life pattern, or feel shackled by knowing about the life of the person from whom they were cloned. Those on both sides mostly agree that, based on the poor success rate in animal cloning and the potential health risks to mother and child, on safety grounds it would be unethical to attempt human cloning currently (Kass, 1998: 694–695; Robertson, 1998: 1372, 1410–1411 and 1415–1416; Burley and Harris, 1999: 110; de Melo-Martín, 2002: 248–250; Harris-Short, 2004: 333 and 344; Tannert, 2006: 239; Mameli, 2007: 87; Morales, 2009: 43; Shapsay, 2012: 357; The Ethics Committee of the American Society for Reproductive Medicine, 2012: 804–805; Wilmut, 2014: 40–41).

Many countries have banned reproductive and/or therapeutic cloning. In most cases, their laws refer to somatic cell nuclear transfer rather than cloning more generally and thus newer technologies are not covered (Lo *et al.*, 2010: 16). Several international and regional measures also prohibit human reproductive cloning: UNESCO’s 1997 *Universal Declaration on the Human Genome and Human Rights* (UNESCO, 1997), the World Health Organization’s resolutions of 1997 and 1998 on the implications of cloning for human health (WHO, 1998), the Council of Europe’s 1998 *Additional Protocol to the Convention on Human Rights and Biomedicine, on the Prohibition of Cloning Human Beings* (Council of Europe, 1998) and the European Union’s 2000 (amended 2007) *Charter of Fundamental Human Rights* (European Union, 2012). As the Council of Europe’s protocol has been ratified by only 23 of its 47 member states, the EU Charter is limited to the enactment of EU law and UNESCO’s declaration is by definition non-binding, none of these represent an absolute ban (Council of Europe, 2016; European Commission, 2016). Hence, at the request of France and Germany, in 2001 the UN General Assembly began to deliberate on a binding treaty to prohibit human reproductive cloning. Four years of dispute and discord followed. Some states were concerned that an embargo on reproductive cloning specifically would implicitly endorse therapeutic or research cloning, whilst those wishing to pursue therapeutic cloning could not support a holistic ban. With agreement on a binding convention seemingly elusive, the General Assembly opted for a non-binding declaration. The *United Nations Declaration on Human Cloning* was duly adopted on 8 March 2005, but not unanimously. 84 states voted in favour, 34 voted against and 37 abstained (Arsanjani, 2006; Isasi and Annas, 2006; Cameron and Henderson, 2007). The declaration, rather ambiguously, calls on states to “prohibit all forms of human cloning inasmuch as they are incompatible with human dignity and the protection of human life” (United Nations, 2005). It is considered too weak an instrument to either thwart rogue research or promote legitimate scientific endeavour (Isasi and Annas, 2006: 63; United Nations University Institute of Advanced Studies, 2007: 19).

## The UNESCO Bioethics Programme

The UNESCO Bioethics Programme began in 1993 with the formation of the International Bioethics Committee (IBC), made up of independent experts. An Intergovernmental Bioethics Committee (IGBC), comprising state representatives, followed in 1999. Each committee has 36 members. The IBC meets yearly and the IGBC biennially. Regular joint meetings of the two committees are also held. The IBC has various functions, including promoting bioethics education and reflection on ethical issues. The IGBC’s mandate is to examine the recommendations of the IBC and report back to the Director-General of UNESCO (UNESCO, 1998). The IBC works on the basis of 2-year Work Programmes (human cloning, for example, featured in the 2008–2009 and 2010–2011 programmes), with reflections on particular topics being drafted by specially appointed Working Groups, comprising a small number of IBC members, over the 2-year cycle. Each Group presents their work-in-progress at IBC and IGBC meetings and takes the views expressed at these meetings into account in their final reports.

Scholars from both within and without the Bioethics Programme have analysed its efficacy as a forum for ethical debate and standard-setting.^1^ These analyses have mostly focused on the negotiation of the 2005 *Universal Declaration on Bioethics and Human Rights*. The interest-based bargaining often seen within intergovernmental organizations led to vague wording on beginning and end of life issues and risk assessment, while controversial issues such as sex selection, gene therapy and stem cell research were left out entirely, as states could not reach a consensus on these (Schmidt, 2007; Langlois, 2013). UNESCO claims that its status as an intergovernmental body differentiates it from ethics institutions outside of the UN like the World Medical Association, a professional body (ten Have, 2006: 342). However, there has been a lack of buy-in from the global bioethics community, particularly academics, who have questioned the expertise and representativeness of the IBC (Cameron 2014: 237 and 240). The lack of enforcement power of the 2005 declaration, as a non-binding instrument, has also been noted. Yet Cameron (2014: 252 and 261) argues that declarations have advantages over conventions, because of their reliance on moral persuasion and their inclusivity in comparison to conventions, which are only binding on those states that accede to them. UNESCO suffered a major setback in 2011, when the United States withdrew funding in light of Palestine’s admittance as a member state, a cut of 22 per cent of the operational budget (UNESCO, 2011e; UNESCO, 2013a).^2^ The Bioethics Programme has emerged relatively unscathed, however, as its budget allocation has largely been protected (UNESCO, 2013c; UNESCO, 2016a).

## The human cloning debate at UNESCO 2008–2011

At the request of then Director-General of UNESCO, Koïchiro Matsuura, in 2008 the IBC decided to investigate the possibility of a convention on human cloning and appointed a Working Group on Human Cloning and International Governance (UNESCO, 2009a: 1–2). This was a response to the publication of a report the previous year by the United Nations University’s Institute of Advanced Studies, entitled *Is Human Reproductive Cloning Inevitable: Future Options for UN Governance*. The Working Group was tasked with reviewing “whether the scientific, ethical, social, political and legal developments on human cloning in recent years justify a new initiative at international level”, rather than examining the ethics and science of human cloning *per se* or drafting a legal text (UNESCO, 2008a: 1). The IBC and IGBC meetings where human cloning was discussed took place as follows: (Table 1)

The Working Group’s first report was an interim report, published in September 2008. It recommended a new, binding international convention to ban human reproductive cloning (UNESCO, 2008b: 4). The report was discussed the following month by the IBC and IGBC (the IBC met for 2 days by itself and then jointly with the IGBC for 2 days), where it was given an ambivalent reception. Many participants did not believe there had been sufficient change in national positions to avoid a repetition of the fractious debate and unsatisfactory outcome at the UN General Assembly a few years before. On the other hand, some delegates underlined the potential utility of a convention for those developing countries yet to legislate on cloning (UNESCO, 2010a: 6 and 12). In response to these discussions, the Working Group was more cautious in its final report of June 2009. Judging that the introduction of a new international normative instrument would be premature, it recommended increased global dialogue as an alternative (UNESCO, 2009a: 7). This suggestion was commended by the IGBC at its July 2009 meeting, with several participants noting that developing countries that do not have “a well-developed national bioethics infrastructure” would benefit particularly from international level debate (UNESCO, 2009b: 4).

The cloning mandate continued into the next Work Programme of 2010–2011. After discussion at its November 2009 meeting and on the advice of the IGBC, the IBC instructed an expanded Working Group to continue its work on cloning by examining three issues: (a) the ethical impact of terminology (b) dissemination activities and (c) regulation of human reproductive cloning (including by moratorium). The Working Group duly delivered a draft report to the IBC and joint IBC–IGBC meetings of October 2010. On options for regulation, it found that a more robust instrument on human reproductive cloning than existed currently was needed, such as an international convention or moratorium (UNESCO, 2010b: 1 and 6). The reception from the IBC and IGBC was again mixed, as reported by the UNESCO website:

IBC members were unequivocal in expressing concern that the recent scientific developments have raised a need for a binding international legal instrument. However, feedback by Member States of IGBC was indicative that the political hurdles that have prevented the realization of such instrument in the past are still in place. [*sic*] (UNESCO, 2016b)

As noted in the official record of the IBC-only meeting, members considered it “imperative” that binding international law to ban human reproductive cloning be put in place (UNESCO, 2011d: 6). By contrast, within the joint IBC–IGBC meeting that followed, the US delegation was perplexed as to why the possibility of a convention was “back on the table”, after it had seemingly been rejected in the 2008–2009 Working Group’s final report. It advocated ongoing dialogue instead, alongside support for states developing national regulations on cloning. Germany and Brazil also backed the status quo, prompting one IBC member to ask why in 2010 they believed a convention to be premature, when in 2001, the year the idea was first put to the UN, they had thought one timely. Meanwhile, some developing countries stated their desire for a convention on cloning (but not necessarily a prohibitive one) (personal observations, Joint Session of the IBC and IGBC, October 2010). Given the diversity of views, it was left that the IGBC would “thoroughly examine the issue” at its next session (to be held in September the following year), after the IBC, via the Working Group, had finalized its report (UNESCO, 2016b).

The IBC held its next meeting in May–June 2011, at which the Working Group presented a draft “final statement” rather than a finalized version of the draft report of the previous year. This statement repeated the recommendations of the 2010 draft report, emphasizing that developing countries that do not have national regulations on human reproductive cloning are in particular need of a binding international convention or moratorium. In addition, it suggested that “technical manipulations of human embryo, either for research or therapeutic purposes” [*sic*] (that is, what is commonly known as therapeutic or research cloning) should carry on being regulated at domestic level, in accordance with social, historical and religious contexts (UNESCO, 2011b: 3). The IBC chose not to adopt the statement because of the now “divergent positions” of its members on both the ethics and governance of cloning (UNESCO, 2011c: 4). At the ethical level, some members were not convinced that the potential for detrimental genetic determinism was a strong enough argument against reproductive cloning, whilst at the political level, some felt the committee could make little progress while consensus among states remained elusive (personal observations, Eighteenth Session of the IBC, May–June 2011).

At the IGBC’s September 2011 meeting, the outgoing IBC Chair reported on his committee’s activities. With regard to the cloning debate, he explained that despite some members having wanted to go to a vote on whether to adopt the Working Group’s draft statement, he had opposed this, because the IBC had always operated by consensus in the past. He also expressed his belief that consensus on a ban will always be impossible to achieve, because at its core the issue is philosophical rather than scientific, concerning the status of the early embryo. IGBC delegations agreed for the most part, the United States, Austria and Denmark echoing IBC members in predicting that further efforts to reach an agreement on regulation would prove fruitless (personal observations, Seventh Session of the IGBC, September 2011). The official conclusions of the meeting noted the topic’s ongoing importance, but also the absence of any consensus among both states and IBC members. Hence the IGBC merely called on UNESCO “to continue to follow the developments in this field in order to anticipate emerging ethical challenges” (UNESCO, 2011a: 3). Subsequently, the 2012–2013 IBC Work Programme consigned cloning to monitoring by a few IBC members, who were in turn to report any significant developments in the field to the committee and thereby the Director-General of UNESCO (UNESCO, 2016f).

After 4 years of work and discussion, then, UNESCO’s inability to come to a consensus on whether or not a convention to ban human reproductive cloning would be desirable meant that a decision against a convention was made by default. The Working Group’s draft final statement of 2011 had concluded, “The current non-binding international regulations cannot be considered sufficient in addressing the challenges posed by the contemporary scientific developments and to safeguard the interests of the developing countries that still lack specific regulations in this area” (UNESCO, 2011b: 3). If this is the case, UNESCO’s failure to meet the need identified by its Working Group is problematic, as there is a governance gap.

## 2014–2015 developments

In its 2014–2015 Work Programme the IBC revisited the topic of human cloning as part of its wider efforts to update its earlier work on the human genome and human rights. The June 2015 draft report of the Working Group appointed to this task reiterated the need for a ban on human reproductive cloning. It also called for “a global forum of scientists and bioethicists, under the auspices of the United Nations” to investigate what the consequences of new genomic technologies might be and stated, “The United Nations should be responsible for making fundamental normative decisions. The precautionary principle should be respected, ensuring that substantial consensus of the scientific community on the safety of new technological applications be the premise for any further consideration” (UNESCO, 2015b: 25–27).

The IGBC, on reviewing this draft report at its July 2015 meeting (Ninth Session), found the IBC’s recommendations to be “pertinent and timely” (UNESCO, 2015a: 2). This was in marked contrast to the comments by some of its members a few years before that a ban on human reproductive cloning would be “premature” (UNESCO, 2009a: 7). Perhaps wary of ceding “territory”, the IGBC stressed that UNESCO was the appropriate forum for discussion of a ban. In the official conclusions of the meeting, it also invited the Secretariat of the Bioethics Programme to “collect and compile existing legal models, case studies and best practices” on cloning and other issues relating to the human genome addressed in the report (UNESCO, 2015a: 2–3). The draft was revised in light of the IGBC’s comments and then discussed and revised again at the IBC’s 22^nd^ Session in October 2015. The final version—*Report of the IBC on Updating Its Re*fl*ection on the Human Genome and Human Rights*—states that the UN should be responsible for fundamental normative decisions “through its several agencies and bodies and other possible procedures of consultation and evaluation” rather than a new global forum. It also asserts UNESCO’s position as a key player in the bioethics community, adding that, in terms of any revisions to existing declarations, “First of all, this is a task to perform for UNESCO, building on its well-established, pivotal role as a global forum for global bioethics” (UNESCO, 2015c: 27–29).

The report addresses several issues that fall under the banner of the human genome and human rights, not just cloning. Nevertheless, cloning is prominent. The Executive Summary includes an “open list” of recommended actions for states and governments. The first item is: “Produce an international legally binding instrument to ban human cloning for reproductive purposes”. There are also recommendations for scientists and regulatory bodies, who are to “renounce the pursuit of spectacular experiments that do not comply with the respect of fundamental human rights” (UNESCO, 2015c: 3–4). The main text expands on this, to state that such experiments should be discouraged (by not being allocated public funds, for instance) and in some cases prohibited, where there is no medical justification and a risk to safety. That this refers to cloning is made explicit, as follows: “Research on the possibility of cloning human beings for reproductive purposes remains the most illustrative example of what should remain banned all over the world” (UNESCO, 2015c: 26). More generally, the report advocates a conservative approach to decision- and law-making that may be particularly relevant to human embryonic stem cell research, or “therapeutic cloning”. It encourages the adoption of legislation at international and national levels that is “as non-controversial as possible, especially with regard to the issues of modifying the human genome and producing and destroying human embryos”, to respect differing sensitivities and cultures (UNESCO, 2015c: 3 and 6).^3^ With regard to developing countries, the report acknowledges that they may not have major access to new genomic technologies in the near future, but recommends that LMIC (low and middle income country) governments develop national policies on genomics “within the context of their national economic and sociocultural uniqueness” (UNESCO, 2015c: 29). The report also makes recommendations for “all actors of civil society”, including the media, educators and businesses. The former are to “avoid any sensationalism”, whilst the latter are not to chase profit by operating in countries with weak regulations (UNESCO, 2015c: 3–4).

## Analysis

Hofferberth (2015: 616) is critical of the assumption that “global problems are tractable and solutions feasible if actors will only come and work together to solve them”. As shown above, some members of the IBC and IGBC believed that the reason why they failed to reach consensus during the first 4 years of debate on human cloning (2008–2011) was the inherently irresolvable nature of the problem itself. But other controversial areas, such as business and human rights, have not proved immune to recent efforts towards policy and norm convergence (Ruggie, 2014: 6). Another possible explanation for the failure, then, is that the legal and organizational structures directing the deliberation did not lend themselves to consensual decision-making. In the early 2000s the UN General Assembly had found that the old model of state-based treaty negotiation did not work for human cloning, when it failed to agree on a convention and chose a non-binding declaration instead. UNESCO’s experience was similar, although it was not negotiations on treaty content that failed, but the preceding stage of deciding whether or not to attempt to draft a treaty. In raising the possibility of a convention in 2008, UNESCO was going against the emerging trend within global governance towards voluntary rather than binding regulation, combined with capacity building. Germany, for example, which was one of the states that originally espoused the idea of a human cloning convention at the UN in 2001, now looks for other, less rigid means by which the goals of a proposed treaty can be reached (Pauwelyn *et al.*, 2014: 739). Within UNESCO, as in other intergovernmental organizations, it is states that make the final decisions, so even if in 2011 the IBC (made up of independent experts) had continued to insist on the desirability of a convention, it would only have had the power to recommend to member states that they take the idea forward.

Pauwelyn *et al.* (2014: 734) advocate “thick stakeholder consensus” over the “thin state consent” that is the hallmark of the old hierarchical approach to governance. As a treaty could be based on back-room deals between undemocratic states and yet be recognized as international law, they argue that formality is no guarantee of legitimacy, if the latter is assessed in terms of inclusiveness and effectiveness rather than tradition. Rather, the process by which agreement is reached is crucial, as well as the outcome. Careful, open and expert deliberation can lead to high quality outputs, which may or may not be legally binding (Pauwelyn *et al.*, 2014: 748–749). One way to achieve both process and output would be to loosen UNESCO’s understanding of “consensus”. By sticking to a rigid definition of consensus at its 2011 meeting, the IBC effectively gave each member a veto. Pauwelyn *et al.* (2014: 754–755) contrast this type of arrangement with the “standards world” (that is, the International Organization for Standardization and the International Electrotechnical Commission), which sits outside the intergovernmental system. Here, where governance is seen to be nimbler and more flexible than in traditional governance settings, “consensus” means that “the views of all parties concerned must be taken into any account and an attempt must be made to reconcile conflicting arguments”, so that general agreement can be reached. This level of consensus might be a more realistic target for the IBC and IGBC, enabling them to move forward.

One problem the Bioethics Programme has faced consistently is lack of time for in-depth discussion. At the IBC meeting in May–June 2011, for instance, the public session devoted to cloning lasted little more than an hour (although the committee later continued its discussions in a private meeting). This was not unusual. At the IGBC’s September 2013 meeting (Eighth Session), which reviewed 20 years of the Bioethics Programme, one delegate stated that their government would stop funding their attendance at such meetings unless more time were given to dialogue and papers were sent out early enough for delegates to consult with the relevant ministries on what position they should take (personal observations, Eighteenth Session of the IBC, May–June 2011 and Eighth Session of the IGBC, September 2013^4^). The Bioethics Programme has already started to implement such changes. More time was allocated to each discussion topic at the IBC and joint IBC–IGBC meetings of September 2014 than at previous sessions, an online forum for past and present IBC members has been established and concept notes to invite written comments from the IGBC on the IBC’s work ahead of meetings have been introduced (UNESCO, 2015d: 2 and 17).

If deliberations were to emulate recent innovations in other intergovernmental fora, they might be improved further. After its disappointing Copenhagen round in 2009, the Conference of the Parties to the *United Nations Framework Convention on Climate Change* has moved from formal treaty negotiations that encouraged bargaining and confrontation to workshops and roundtables designed to foster knowledge exchange. This has resulted in “positive competitive dynamics” among states wishing to be leaders in the field of climate change mitigation (Rietig, 2014: 372–374). Other stakeholders have also been given a stronger voice; the Paris conference of 2015 made space for NGOs, businesses and cities to share best practices. Furthermore, the *Paris Agreement* of December 2015 takes a bottom-up approach, in that it is based on Intended Nationally Determined Contributions (pledged targets and actions) by individual states (Busby, 2016: 3, 4 and 7). Similarly, after the UN failed to adopt both a code of conduct and a set of norms on business and human rights after several years of trying, it piloted a different standard-setting method. Based on a series of site visits to firms and communities, extensive research and testing of key proposals through feasibility studies, pilot grievance mechanisms and scenario-based exercises, as well as multistakeholder consultations, the *Guiding Principles on Business and Human Rights* were endorsed by the Human Rights Council in 2011 and have since been adopted by several other bodies, including business associations. Ruggie (2014: 5–6 and 10), who directed the consultation process, claims that producing the guiding principles through this “polycentric governance” enabled them to achieve the “thick” consensus advocated by Pauwelyn *et al*.

Ruggie (2014: 10) argues that conceptual arguments must be supported by experiential ones if they are to persuade people of the need for change. The cloning debate is necessarily conceptual, as while questions over safety prevail there is no way to experience cloning to see whether fears (about autonomy and individuality, for example) are founded or unfounded. The closest proxies are animal cloning and twin studies. Yet sharing of national regulations and policies on cloning via workshops and roundtables and scenario-based exercises involving potential stakeholders would be feasible. Similar exercises (collating examples of legal frameworks, best practices and case studies) were suggested by the IGBC in their response to the IBC’s 2015 draft report on the human genome and human rights. Such activities could meet developing countries’ needs for something on which to base national cloning legislation, identified by all three IBC Working Groups (2008–2009, 2010–2011 and 2014–2015), by alternative means to a binding international convention, the latest recommendations of the IBC on this (and the IGBC’s endorsement of them) notwithstanding. Continuing to develop the Bioethics Programme’s deliberative format, away from short, formal discussions within committees towards more in-depth information exchange between a broader range of stakeholders, bottom-up pledges of action and development of best practice through feasibility studies, may not result in a decision to begin negotiating a treaty (or even a softer declaration), but could lead to a set of resources and commitments that might prove equally effective in promoting ethical behaviour on the part of states and other actors. An added benefit would be that this type of less legalistic, more flexible deliberative output could be more easily adapted and developed to take account of future scientific advances (Pauwelyn *et al.*, 2014: 742–743). Even if UNESCO were to decide to follow the IBC’s 2015 recommendation to pursue the elaboration a further international legal instrument on human cloning, adopting these measures could result in a qualitatively stronger instrument than the *Universal Declaration of Bioethics and Human Rights*, for example, as there would be less interest-based bargaining and more buy-in from stakeholders.

## Conclusion

When intergovernmental organizations are unable to agree on a form of binding international law such as a convention, they sometimes settle for a declaration, which is less demanding of states. This occurred at the UN in 2005, when the General Assembly could not resolve its members’ differences on what the content and reach of a convention on human cloning should be. Declarations have been the preferred option for UNESCO’s Bioethics Programme in the past, as the drafting period is usually shorter than for a convention and the final product is more likely to inspire consensus, partly because it will be seen to be more flexible and less onerous than a binding piece of legislation (Langlois, 2013: 65–66). But this was not a viable path for UNESCO when it came to the regulation of human cloning, because an international declaration—the *United Nations Declaration on Human Cloning* of 2005—already existed. The Bioethics Programme thus broke with previous practice and began to investigate the possibility of a convention on cloning in 2008. There was tension between IBC and IGBC members over whether a convention would be desirable, with the former (the independent experts) supporting a ban on human reproductive cloning and the latter (representing states) concerned that negotiations would simply revisit the disagreements of the UN General Assembly debates of a few years before. Ultimately, with consensus within and between the two committees proving elusive, the idea of a cloning convention dropped from their agendas in 2012.

The idea was taken up again in 2014, as part of the IBC’s work on the human genome. We can only speculate as to why the IGBC of 2015 was keener on a ban on human reproductive cloning than the IGBC of 2008–2011. The United States was no longer a member, but Germany and Brazil still were (UNESCO, 2016c). It could be that, since the first human therapeutic (or research) cloning via somatic cell nuclear transfer took place in 2013 (Tachibana *et al.*, 2013), human reproductive cloning has moved from the realms of science fiction to real possibility in the eyes of policy-makers. Or the changes to the deliberative format at IBC and IGBC meetings introduced in 2014, such as presession concept notes and longer discussions, may have engendered greater consensus between the two committees. Yet, despite this consensus, there has been no move on the part of UNESCO to start to develop a treaty. In past standard-setting endeavours, an IBC Working Group has done the initial drafting, but the IBC Work Programme of 2016–2017 makes no mention of human cloning (UNESCO, 2016d).

For those states that have yet to formulate national regulations or policies on human cloning, the continued lack of clear guidance at international level may be particularly unhelpful. Thus better global governance in this area is needed. In its 2015 report on the human genome and human rights, the IBC fell somewhere between old and new forms of global governance. There was a strong call for an international binding instrument on human reproductive cloning, to be produced by states and governments, but there were also recommended actions and principles for a broad range of stakeholders, including national governments, scientists, the media, educators and corporations. The science and politics of human cloning have moved on since 2011, when states’ positions were seemingly intractable. Were the Bioethics Programme to mirror successful moves in other fora, such as the Conference of the Parties to the *United Nations Framework Convention on Climate Change* and the Human Rights Council, towards knowledge sharing, scenario-based exercises and action pledges involving a wide range of stakeholders, a robust global governance framework for human cloning—whether a legally binding instrument or something more flexible—might be achievable.

## Figures and Tables

**Table 1:** IBC and IGBC meetings where cloning was discussed, 2008–2011

Date	Event
28 to 31 Oct 2008	Fifteenth Session of the IBC (2 days); Joint Session of the IBC and IGBC (2 days) (Paris)
9 to 10 Jul 2009	Sixth Session of the IGBC (Paris)
23 to 25 Nov 2009	Sixteenth Session of the IBC (Mexico City)
26 to 29 Oct 2010	Seventeenth Session of the IBC (2 days)*,; Joint Session of the IBC and IGBC (2 days)* (Paris)
31 May to 2 Jun 2011	Eighteenth Session of the IBC* (Baku)
5 to 6 Sep 2011	Seventh Session of the IGBC* (Paris)

* Meetings attended and observed by the author.

## Footnotes

1 See, for example, Macpherson CC (2007) Global bioethics: Did the universal declaration on bioethics and human rights miss the boat? *Journal of Medical Ethics*; **33** (10): 588–590; Snead CO (2009) Bioethics and self-governance: The lessons of the Universal Declaration on Bioethics and Human Rights. *Journal of Medicine and Philosophy*; **34** (3): 204–222; Kirby M (2010) Health care and global justice. *Singapore Academic of Law Journal*; **22** (special ed. 2): 785–800; Langlois A (2013) *Negotiating Bioethics: The Governance of UNESCO*’*s Bioethics Programme*. Routledge: Abingdon; Cameron NM de S (2014) Humans, rights, and twenty-first century technologies: The making of the Universal Declaration on Bioethics and Human Rights. *Journal of Legal Medicine*; 35(2): 235–272.

2 After 2 years of withholding its dues, the United States lost its General Conference voting rights in 2013 (UNESCO, 2013b). It has not had a seat on the IGBC since 2011.

3 This cautious, conservative approach is in marked contrast to the advice John Harris, an ethicist, gave at the celebration event to mark the twentieth anniversary of UNESCO’s Bioethics Programme, held at UNESCO headquarters in Paris in September 2013. He stated, “There is the danger—and this is the note on which I wish to end—the danger presented by the precautionary principle itself, which I also believe is one of the biggest dangers facing society and humanity. People often believe that there is some moral imperative to be ultra-cautious in permitting new research, particularly in the general field of genetics. And this caution has also been very true of UNESCO’s approach. However, it is not unusual to find this so-called precautionary principle being invoked in circumstances in which it is far from clear in which direction, if any, caution lies. We cannot know in which direction caution lies without having some rational basis for establishing the scale of likely dangers from pursuing particular programmes of research and innovation and comparing those with the on-going costs of failing to pursue that research to a conclusion. … I hope UNESCO will avoid the terrible mistake it made in Article 11 of the declaration on the human genome of saying, without argument or evidence, without a scintilla of support, that human cloning was contrary to human dignity and must be outlawed. We’re going to have to rethink that. We’re going to need human cloning as one technique among many others. … We need to rethink our prejudices. We need to be slow to outlaw technology. That doesn’t mean we shouldn’t do it, but we should also do so on the full consideration of the evidence and the argument and never simply because it would be cheap, easy and popular.” (Transcribed from the live webcast of the event, 5 September 2013. Available at: mms://stream.unesco.org/live/room_11_en.wmv. Last accessed 5 September 2013.)

4 The author listened to the Eighth Session of the IGBC in September 2013 via live webcast. (mms://stream.unesco.org/live/room_11_en.wmv. Last accessed 5 September 2013.)
